# Insecticidal and Biting Deterrent Activities of *Magnolia grandiflora* Essential Oils and Selected Pure Compounds against *Aedes aegypti*

**DOI:** 10.3390/molecules25061359

**Published:** 2020-03-17

**Authors:** Abbas Ali, Nurhayat Tabanca, Betul Demirci, Vijayasankar Raman, Jane M. Budel, K. Hüsnü Can Baser, Ikhlas A. Khan

**Affiliations:** 1 National Center for Natural Products Research, The University of Mississippi, Oxford, MS 38677, USA; nurhayat.tabanca@usda.gov (N.T.); vraman@olemiss.edu (V.R.); ikhan@olemiss.edu (I.A.K.); 2 United States Department of Agriculture, Agricultural Research Service (USDA-ARS), Subtropical Horticulture Research Station (SHRS), Miami, FL 33158, USA; 3 Department of Pharmacognosy, Faculty of Pharmacy, Anadolu University, 26470 Eskisehir, Turkey; betuldemirci@gmail.com; 4 Departamento de Ciências Farmacêuticas, Universidade Estadual de Ponta Grossa (UEPG), Ponta Grossa, PR 84030-900, Brazil; janemanfron@hotmail.com; 5 Department of Pharmacognosy, Faculty of Pharmacy, Near East University, 99138 Nicosia, Northern Cyprus; khcbaser@gmail.com

**Keywords:** Magnoliaceae, GC-FID, GC-MS, mosquito control, 1-decanol, 1-octanol, larvicidal activity, deterrent, biopesticides

## Abstract

In our natural products screening program for mosquitoes, we tested essential oils extracted from different plant parts of *Magnolia grandiflora* L. for their insecticidal and biting deterrent activities against *Aedes aegypti*. Biting deterrence of seeds essential oil with biting deterrence index value of 0.89 was similar to *N*,*N*-diethyl-3-methylbenzamide (DEET). All the other oils were active above the solvent control but the activity was significantly lower than DEET. Based on GC-MS analysis, three pure compounds that were only present in the essential oil of seed were further investigated to identify the compounds responsible for biting deterrent activity. 1-Decanol with PNB value of 0.8 was similar to DEET (PNB = 0.8), whereas 1-octanol with PNB value of 0.64 showed biting deterrence lower than 1-decanol and DEET. The activity of 1-heptanol with PNB value of 0.36 was similar to the negative control. Since 1-decanol, which was 3.3% of the seed essential oil, showed biting deterrence similar to DEET as a pure compound, this compound might be responsible for the activity of this oil. In in vitro A & K bioassay, 1-decanol with MED value of 6.25 showed higher repellency than DEET (MED = 12.5). Essential oils of immature and mature fruit showed high toxicity whereas leaf, flower, and seeds essential oils gave only 20%, 0%, and 50% mortality, respectively, at the highest dose of 125 ppm. 1-Decanol with LC_50_ of 4.8 ppm was the most toxic compound.

## 1. Introduction

Insect disease vectors transmit many disease pathogens and are important in global public health. *Aedes aegypti* (L.) and *Ae. albopictus* (Skuse) are the primary and secondary vectors of Zika and dengue as well as other viruses [1]. The use of synthetic insecticides in mosquito control has proven to be one of the major approaches for the prevention and reduction of mosquito-borne disease incidence [2]. Insect repellents also play an important role in the reduction of disease incidence by preventing infected mosquitoes from biting humans [3]. Moreover; repellents have always been used against host-seeking vectors as they provide immediate; localized; personal protection. *N*,*N*-Diethyl-3-methylbenzamide (DEET) has been in use for more than 60 years and is the gold standard to which all repellents are measured in the marketplace [4]. The discovery of novel insecticides and repellents against disease vectors from non-toxic and biodegradable plant sources continues to be the focus of recent research efforts [5,6,7,8].

*Magnolia grandiflora* L. (Magnoliaceae) is a large evergreen tree native to North America [9] that has medicinal and ornamental values. Medicinal use of various parts of *M. grandiflora* is reported in American Indian medicine and also listed as a bitter tonic and antimalarial. Several biologically active compounds have been reported from *Magnolia* species [10,11,12]. As a part of our natural product screening program for mosquitoes, we tested essential oils from various parts of *M. grandiflora* for their larvicidal and biting deterrent activities. This paper reports insecticidal and biting deterrent activities of essential oils and select pure compounds from various parts of *M. grandiflora* against yellow fever mosquito, *Aedes aegypti*.

## 2. Results

Water-distilled essential oils of the leaves, flowers, immature fruits, mature fruits, and seeds of *M. grandiflora* were analyzed by GC-FID and GC-MS. Chemical compositions of the essential oils and total ion current (TIC) chromatogram are given in Table 1 and Figure S1 (Suplementary Material). Chemical profiles of the oils varied among essential oils. Sesquiterpene hydrocarbons (31.5%) were dominant in the seeds and immature fruit essential oils (32%) whereas the leaf and flower oils were rich in monoterpene hydrocarbons (30.9% and 43.8%, respectively). Oxygenated monoterpenes (36.9%) were the major components of the mature fruit oil followed by monoterpene hydrocarbons (15.2%), sesquiterpene hydrocarbons (15.5%), and oxygenated sesquiterpenes (16.2%). The α- and β-pinenes and 1,8-cineole were the major contents of leaf, flower, immature fruit, and mature fruit whereas these compounds were either very low or absent in seed essential oil (Table 1). The seed oil was differentiated from other essential oils because of the presence of fatty acid; hexadecanoic acid (2.9%) and fatty acid esters (2.2%). The saturated aliphatic esters (10.9%) and two phenolic compounds; methyl chavicol (2.6%) and eugenol (1.3%) were also only found in the seed essential oil. Major compounds, α- and β-pinenes and 1,8-cineole present in leaf, flower, immature fruit, and fruit essential oils were either in very low concentration or absent in seed essential oil (Table 1). Fatty acids and esters (5.1%) were high and aliphatic esters (10.9%) were present only in the seed essential oil. Hexadecanoic acid (2.9%), 1-decanol (3.3%), 1-octanol (6.2%), and 1-heptanol were also present only in the seed essential oil. 

**Figure 1:**
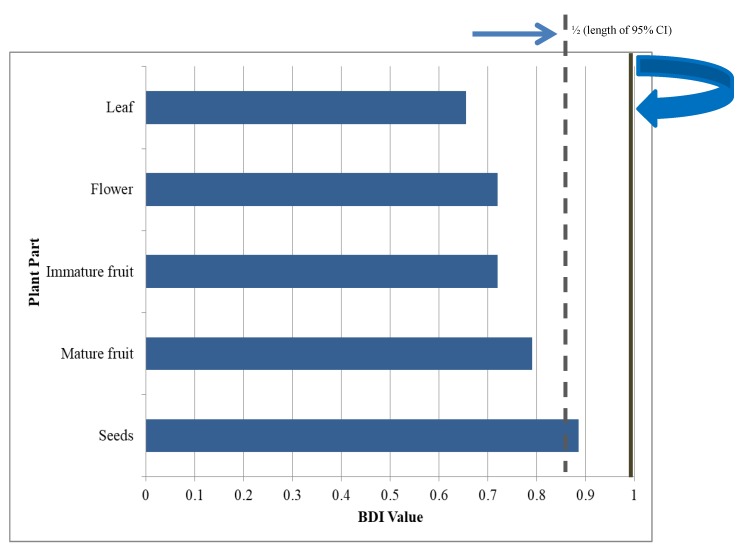
Mean BDI (± SEM) values of the essential oils from different parts of *M. grandiflora* against *Ae. aegypti.* All the essential oils were tested at 10 µg/cm^2^ whereas DEET (*N*,*N*-diethyl-3-methylbenzamide) was tested at 25 nmol/cm^2^ and ethanol a solvent control. A BDI value greater than 0 indicates biting deterrence relative to ethanol and a BDI value not significantly different from 1 shows deterrence similar to DEET.

**Figure 2:**
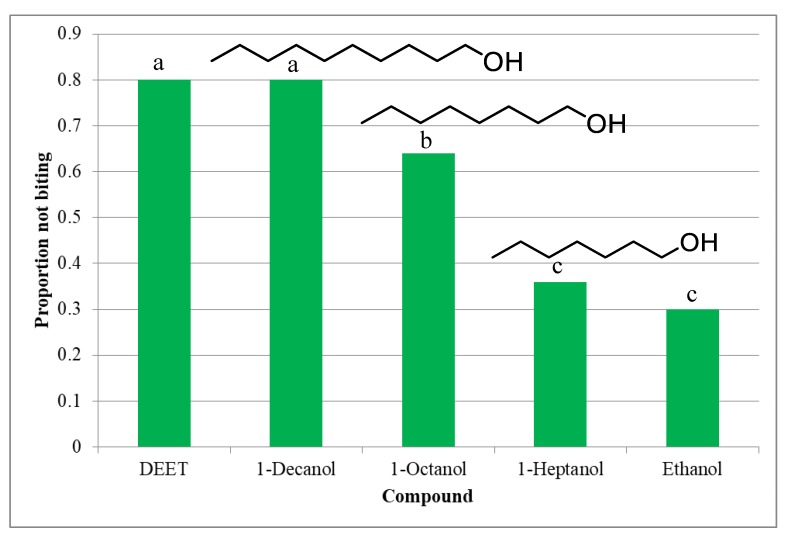
Mean proportion not biting values of the essential oils from different parts of *M. grandiflora* against *Ae. aegypti*. Essential oils were tested at 10µg/cm^2^ while DEET at 25 nmol/cm^2^ was tested as a positive control. Mean proportions sharing the same letter are not significantly different.

The essential oils obtained from five different plant parts of *M. grandiflora* were investigated for their biting deterrent activity against *Ae*. *aegypti*. All the essential oils showed biting deterrence above the negative control. Seeds essential oil produced significantly higher biting deterrence than the essential oils from the other parts (Figure 1). Seed essential oil with high minimum effective dose BDI value (0.89) showed biting deterrent activity similar to DEET whereas all the other essential oils had activity lower than DEET. 1-Decanol with PNB value of 0.8 showed biting deterrence similar to DEET (PNB = 0.8) whereas the activity of 1-octanol was above negative control but lower than DEET and 1-decanol (Figure 2).

**Figure 3:**
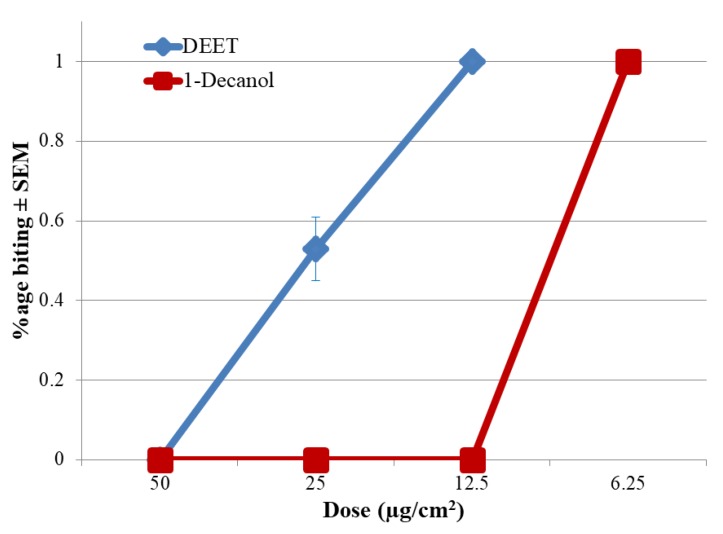
Data are %age (mean ± SEM) females biting. Minimum effective dosage (MED) values in this bioassay were ≤ 1% biting in 1 min which is two females out of 200 in this cage.

Biting deterrence of 1-heptanol was similar to the negative control. 1-Decanol with 3.3% of seed essential oil appears to be the major compound responsible for the biting deterrent activity of the seed essential oil. In in vitro A & K bioassay, MED value of 1-decanol (6.25) was lower than DEET (12.5) which indicated better repellency of 1-decanol than DEET (Figure 3).

The toxicity of essential oils from *M. grandiflora* against 1-d-old larvae of *Ae. aegypti* is given in Table 2. In initial screening, essential oils of immature and mature fruits showed high toxicity whereas leaf, flower, and seeds essential oils gave only 20%, 0%, and 50% mortality, respectively, at the highest screening dose of 125 ppm. Therefore dose-response bioassays were not conducted on leaf, flower, and seeds essential oils. Immature fruit and mature fruit essential oils with LC_50_ values of 49.4 and 48.9 ppm, respectively at 24-h post-treatment showed similar toxicity. 

Pure compounds 1-decanol, 1-octanol, and 1-heptanol, present in seed essential oil were also screened for larvicidal activity. Both 1-decanol and 1-octanol were active in screening bioassays whereas 1-heptanol did not show any mortality at the highest dose of 125 ppm. 1-Decanol and 1-octanol were further evaluated to observe the dose response. 1-Decanol with LC_50_ of 4.8 ppm was the most toxic compound followed by 1-octanol (LC_50_ = 34.3 ppm) at 24-h post-treatment. 1-Decanol was very toxic (LC_50_ = 4.8 ppm) as a pure compound, the seed essential oil that contained 3.3% of this compound showed 50% mortality at the highest dose of 125 ppm. 1-Decanol amounted to be 4.07 ppm as a part of seed essential oil at 125 ppm which caused mortality similar to the pure compound. 1-Decanol and 1-octanol amounted to be 4.07 and 7.75 ppm, respectively, as a part of the essential oil dose of 125 ppm. Since the toxicity of 1-octanol as a pure compound was low, the main compound responsible for the toxicity of the seed essential oil appears to be 1-decanol.

## 3. Discussion

Schuhly et al. [13] reported β-elemene as the major compound in the fruit essential oil of *M. grandiflora* which corroborates the finding of this study having β-elemene (6–14%) in all the essential oils of *M. grandiflora* except seed oil. Guerra-Boone et al. [14] reported bornyl acetate (20.9%) as the major compound in *M. grandiflora* leaf oil, however, we did not detect this compound in the leaf oil. Garg and Kumar [15] reported β-caryophyllene as the major compound (34.8%) in flower essential oil whereas only a small amount (1.1%) was detected in the present study. Farag and Al-Mahdy [16] reported variation in the contents of *M. grandiflora* flower oil volatiles obtained through headspace and water distillation techniques indicating the effects of the isolation technique on the yield of different compounds. Such differences in chemical compositions of essential oils are expected and can be attributed to many factors including geographic location, genetic factors, climate, crop stage, harvesting time, and processing method [17,18].

In our previous studies, some of the compounds that were present in these essential oils exhibited very insignificant biting deterrence. α-Phellandrene (BDI = 0.52), (+)-α-pinene (BDI = 0.47), (-)-α-pinene (BDI = 0.41), (+)-β-pinene (BDI = 0.57), (-)-β-pinene (BDI = 0.51), *p*-cymene (BDI = 0.48), *trans-*sabinene hydrate (BDI = 0.61) showed biting deterrent activity lower than DEET. β-caryophyllene and caryophyllene oxide with BDI values of 0.54 and 0.66, respectively, were also significantly lower than DEET at 25 nmol/cm^2^ and these two sesquiterpenes also did not repel mosquitoes up to the highest dose of 1.5 mg/cm^2^ in cloth patch assay [6,19,20,21,22]. Hexadecanoic acid that was only present in seed essential oil was reported to have biting deterrence lower (PNB = 0.72) than DEET [23]. In our previous study, we found that mid-chain length acids (C_10:0_ to C_13:0_) showed the highest biting deterrent activity against Ae. aegypti as compared to short-chain length acids (C_6:0_ to C_9:0_) [22]. The current study reveals similar pattern of medium chain length fatty alcohol (C_10:0_) had higher biting deterrent activity than short-chain length alcohol (C_8:0_). However, we shall work on this hypothesis and confirm the activity toward short, med, and long-chain fatty alcohols. Many methylbutyrates present in the seed essential oil were tested in our screening program and found not active as biting deterrents (Ali personal communications). Since most of the major compounds that were present only in seed essential oil did not show any significant activity, 1-decanol might be the main compound responsible for the biting deterrent activity of *M. grandiflora* seed essential oil.

The toxicity of many natural compounds present in plant essential oils against mosquitoes has been reported in the literature. α-Pinene (LC_50_ = 49.5 ppm), β-pinene (LC_50_ = 35.9 ppm), β-caryophyllene (LC_50_ = 26.0 ppm), and caryophyllene oxide (LC_50_ = 29.8 ppm) were active as larvicides against *Ae. aegypti* whereas 1,8-cineole did not show any mortality at the highest screening dose of 125 ppm [19,23]. Monoterpenes that were present in variable concentrations in these essential oils showed larvicidal activity. These higher percentages of monoterpenes (α- and β-pinenes) in combination with other compounds may be responsible for the high toxicity of immature- and mature fruit essential oils. We will further explore other compounds present in *M. grandiflora* essential oils for their potential as larvicides against mosquitoes. Ethanolic extracts of sarcotesta of the seeds of *M. dealbata* were reported to have 96.4% mortality at 0.1 mg/mL against the Mexican fruit fly (*Anastrepha ludens* Loew) whereas the extracts from the other parts were inactive [24].

## 4. Materials and Methods

### 4.1. Chemicals

Individual compounds such as 1-decanol (CAS # 112-30-1), 1-octanol (CAS# 111-87-5), and 1-heptanol (CAS # 111-70-6) were obtained from the National Center for Natural Products Research (NCNPR) Repository of The University of Mississippi, University, MS, USA Repository. These compounds were previously purchased from Sigma-Aldrich Co., St. Louis, MO, USA. 

### 4.2. Plant Materials

**Figure 4:**
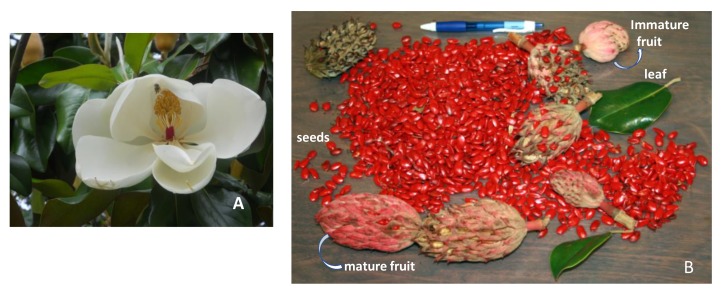
Study material. **A**: flowers; **B**: leaves, immature fruits, mature fruits and seeds. Photos courtesy of V.R.

Whole samples of leaves, flowers, immature and mature fruits, and seeds (Figure 4) were freshly collected from an identified *M. grandiflora* tree at the University of Mississippi campus in 2018. Voucher specimens of all the samples—leaves (NCNPR # 20286), stem-bark (# 20874), flowers (# 20316), immature fruit (# 20871), mature fruit with seeds removed (# 20872), and seeds (# 20873)—were deposited in the Repository of Botanicals at NCNPR, University of Mississippi.

### 4.3. Extraction of Essential Oils

**Figure 5:**
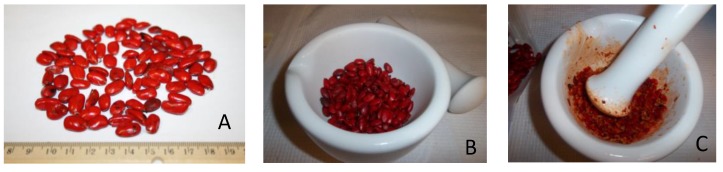
Study material. **A**: Seeds were separated from mature fruits; **B**: seeds were prepared and gently crushed in a mortar and pestle (**C**), and were subsequently hydrodistilled. Photos courtesy of V.R and N.T.

For the extraction of essential oils, leaves, flowers, immature and mature fruits, and seeds of *M. grandiflora* were separately subjected to hydrodistillation for 3 h, using a modified Clevenger-type apparatus. Seeds were crushed in a mortar and pestle before hydrodistillation (Figure 5). The resultant oils were stored in glass vials at 4 ± 0.5 °C with no light. The yields were calculated on a moisture-free basis for mature fruits, flowers, seeds, immature fruits and leaves at 0.02, 0.06, 0.1, 0.1, 1.5% respectively whereas there was no oil present in the stem-bark sample.

### 4.4. GC-MS Analysis

The GC-MS analysis was carried out with an Agilent 5975 GC-MSD system Agilent 5975 (SEM Ltd., Istanbul, Turkey). Innowax FSC column (60 m × 0.25 mm, 0.25 μm film thickness) was used with helium as the carrier gas (0.8 mL/min). GC oven temperature was kept at 60 °C for 10 min and programmed to 220 °C at a rate of 4 °C/min, and kept constant at 220 °C for 10 min and then programmed to 240 °C at a rate of 1 °C/min. The split ratio was adjusted at 40:1. The injector temperature was set at 250 °C. Mass spectra were recorded at 70 eV. The mass ranged from *m/z* 35 to 450.

### 4.5. GC Analysis

The GC analysis was carried out using an Agilent 6890N GC system Agilent 5975 (SEM Ltd., Istanbul, Turkey). The FID detector temperature was 300 °C. To obtain the same elution order with GC-MS, simultaneous auto-injection was done on a duplicate of the same column applying the same operational conditions. Relative percentage amounts of the separated compounds were calculated from FID chromatograms. The analysis results are given in Table 1.

Identification of the essential oil components was carried out by comparison of their relative retention times with those of authentic samples or by comparison of their relative retention index (RRI) to series of *n*-alkanes [25,26]. Computer matching against commercial (Wiley GC/MS Library, MassFinder Software 4.0) and in-house “Başer Library of Essential Oil Constituents” which includes over 3200 genuine compounds with MS and retention data from pure standard compounds and components of known oils as.

### 4.6. Insects

*Aedes aegypti* used in these studies were from a laboratory colony maintained at the Mosquito and Fly Research Unit, Center for Medical, Agricultural and Veterinary Entomology, USDA-ARS, Gainesville, Florida since 1952. We received the eggs and stored them in our laboratory until needed. Mosquitoes were reared to the adult stage by feeding the larvae on a larval diet of 2% slurry of 3:2 beef liver powder (now Foods, Bloomingdale, Illinois) and Brewer’s yeast (Lewis Laboratories Ltd., Westport, CT, USA). The eggs were hatched and reared to the pupal stage in an environment-controlled room at a temperature of 27 °C ± 2 °C and 60 ± 10% RH in a photoperiod regimen of 12:12 (L: D) h. The adults were fed on cotton pads moistened with a 10% sucrose solution placed on the top of screens of 4-L cages.

### 4.7. Mosquito Biting Bioassay

Bioassays were conducted using a six-celled in vitro Klun and Debboun (K & D) module bioassay system developed by Klun et al. [27] for quantitative evaluation of biting deterrent properties of compounds. The K & D system consists of a six-well reservoir with each of the 4 × 3 cm wells containing 6 mL of feeding solution. We used the CPDA-1 ± ATP solution instead of human blood [22]. CPDA-1 and ATP preparations were freshly made on the day of the test and contained a green fluorescent tracer dye (fluorescent water-based tracer “Green”; www.blacklightworld.com) that allowed for the identification of mosquitoes that were fed on the solution. The squashed mosquitoes were observed under black light (FEIT, BPESL15T/BLB 13W 120VAC 60Hz 200mA, Ul#E170906) for feeding. DEET (97% purity *N*,*N*-diethyl-3-methylbenzamide) was used as a positive control (Sigma-Aldrich Co., St. Louis, MO, USA) and ethanol (Fisher Scientific Chemical Co. Fairlawn, NJ, USA) was used as solvent control. Stock and dilutions of all extracts and DEET were prepared in ethanol. All essential oils were evaluated at dosages of 10 µg/cm^2^ and DEET along with the pure compounds was tested at a concentration of 25 nmol/cm^2^.

The temperature of the solution was maintained at 37 °C by using a circulatory bath. The test compounds and controls were randomly applied to six 4 × 3 cm marked portions of nylon organdy strip, which was positioned over the six, membrane-covered wells. A six-celled K & D module containing five 6–15-day-old females per cell was positioned over the six wells, trap doors were opened and mosquitoes allowed access for 3 minutes, after which they were collected back into the module. Mosquitoes were squashed and the presence of green dye (or not) in the gut was used as an indicator of feeding. A replicate consisted of six treatments: four samples, DEET, and ethanol as solvent control. Five replicates were conducted per day using new batches of mosquitoes in each replication.

### 4.8. In vitro A & K Repellent Bioassay

Bioassays were conducted using Ali and Khan (A & K) bioassay system developed by Ali et al. [28] for quantitative evaluation of repellency against mosquitoes. Minimum effective dosage (MED) values in this bioassay were determined using a method described by Katritzky et al. [29]. Briefly, the bioassay system consists of a 30 × 30 × 30 cm collapsible aluminum cage having one penal of clear transparent acrylic sheet with 120 × 35 mm slit through which the blood box containing a removable feeding device was attached. The top of the blood box had a sliding door used to expose the females to the treatment during the bioassay. Rectangular areas of 4 × 7.5-cm were marked on the collagen sheet that matched the measurement of the rectangular liquid reservoirs. Treatments were applied in a volume of 107 µL using a micropipette. Treated collagen was secured on the feeding reservoir containing the feeding solution using a thin layer of grease (Dow Coming Corp., Midland, MI, USA). The feeding device was then pushed inside the blood box and the sliding door was opened to expose the females to the treatment. The numbers of females landing and biting were recorded visually for 1 min. To ensure proper landing and biting, we used 3-4 cages at a time and only one treatment replication of individual compounds was completed in a single cage. The data are presented as %age biting as a function of concentration. MED is ≤ 1% biting out of 200 females in the cage. A total of five replicates were conducted.

### 4.9. Larval Bioassay

Bioassays were conducted using the bioassay system described by Pridgeon et al. [30] to determine the larvicidal activity of essential oils from different parts of *Magnolia grandiflora* against *Ae. aegypti*. Eggs were hatched and larvae were held overnight in the hatching cup in a temperature-controlled room maintained at a temperature of 27 ± 2 °C and 60 ± 10% RH. Five 1-day-old larvae were transferred in each of 24-well tissue culture plates in a 40–50 µL droplet of water. Total of 50 µL of larval diet (2% slurry of 3:2 beef liver powder and brewer’s yeast) and 1 mL of deionized water were added to each well by using a Finnpipette stepper (Thermo Fisher, Vantaa, Finland). All the essential oils and pure compounds were diluted in DMSO. After the treatment, the plates were swirled in clock-wise and counter-clockwise motions and front and back and side to side five times to ensure even mixing of the chemicals. Larval mortality was recorded 24-h post-treatment. Larvae that showed no movement in the well after manual disturbance were recorded as dead. A series of 4-5 dosages were used in each treatment to get a range of mortality. Treatments were replicated ten times for each extract/compound.

### 4.10. Statistical Analyses

Proportion not biting (PNB) was calculated using the procedure described by Ali et al. [22]. As the K & D module bioassay system can handle only four treatments along with negative and positive controls to make direct comparisons among more than four test compounds and to compensate for variation in overall response among replicates, biting deterrent activity was quantified as biting deterrence index (BDI) [22]. The BDI’s were calculated using the following formula:

$$\left[BDI_{i,j,k}\right]=\left[\frac{\begin{array}{ccc} PNB_{i,j,k} & - & PNB_{c,j,k} \end{array}}{\begin{array}{ccc} PNB_{d,j,k} & - & PNB_{c,j,k} \end{array}}\right]$$

where PNB*_i,j,k_* denotes the proportion of females not biting when exposed to test compound *i* for replication *j* and day *k* (*i* = 1–4, *j* = 1–5, *k* = 1–2), PNB*_c,j,k_* denotes the proportion of females not biting the solvent control “*c*” for replication *j* and day *k* (*j* = 1–5, *k* = 1–2) and PNB*_d,j,k_* denotes the proportion of females not biting in response to DEET “*d”*(positive control) for replication *j* and day *k* (*j* = 1–5, *k* = 1–2). This formula adjusts for inter-day variation in response and incorporates information from the solvent control as well as the positive control.

A BDI value of 0 indicates an effect similar to ethanol, while any value greater than 0 indicates biting deterrent effect relative to ethanol. BDI values not significantly different from 1, are statistically similar to DEET. BDI values were analyzed using SAS Proc ANOVA [31]. To determine whether confidence intervals include the values of 0 or 1 for treatments, Scheffe’s multiple comparison procedure with the option of CLM was used in SAS [31]. LC_50_ values for larvicidal data were calculated by using SAS, Proc Probit [31].

## 5. Conclusions

The essential oil of *M. grandiflora* seeds exhibited biting deterrent activity activity similar to DEET. All the major compounds (concentration >1%) except 1-decanol that were present only in seed essential oil were not active biting deterrents which indicated that the major activity of this essential oil might be due to 1-decanol. 1-Decanol also showed promising larvicidal activity. This high activity of 1-decanol indicated the potential of this compound to be developed as an effective mosquito population management tool. Further studies will be continued to evaluate these natural products in different formulations in large scale laboratory bioassays and field trials.

## Figures and Tables

**Table 1:** The chemical composition of essential oils of the *Magnolia grandiflora.*

RRI	Compound	Leaf (%)	Flower (%)	Immature Fruit (%)	Mature Fruit (%)	Seed (%)	IM
1032	α-Pinene	6.3	8.0	4.8	3.8	1.0	RRI, MS
1063	Ethyl 2-methylbutyrate	-	-	-	-	1.5	MS
1076	Camphene	0.1	0.7	1.1	1.6	-	RRI, MS
1100	Isobutyl isobutyrate	-	-	-	-	0.7	MS
1118	β-Pinene	23.0	32.3	12.7	6.9	1.2	RRI, MS
1132	Sabinene	-	0.3	-	-	-	RRI, MS
1151	Propyl 2-methylbutyrate	-	-	-	-	1.0	MS
1174	Myrcene	-	0.4	-	-	0.6	RRI, MS
1176	α-Phellandrene	-	-	-	-	1.1	RRI, MS
1185	Isobutyl 2-methylbutyrate	-	-	-	-	2.4	MS
1198	Isobutyl 3-methylbutyrate	-	-	-	-	1.9	MS
1203	Limonene	1.1	1.0	1.7	1.4	1.0	RRI, MS
1213	1,8-Cineole	4.1	4.4	4.5	12.2	-	RRI, MS
1218	β-Phellandrene	-	-	-	-	7.3	RRI, MS
1241	Butyl-2-methylbutyrate	-	-	-	-	1.3	MS
1246	(*Z*)-β-Ocimene	-	0.8	-	-	-	MS
1280	*p*-Cymene	0.4	0.3	0.8	1.3	5.5	RRI, MS
1290	Terpinolene	-	-	0.5	0.2	-	RRI, MS
1286	2-Methyl butyl 2-methylbutyrate	-	-	-	-	1.3	MS
1299	2-Methylbutyl isovalerate	-	-	-	-	0.8	MS
1429	Perillene	-	-	-	-	0.4	MS
1450	*trans*-Linalool oxide (*Furanoid*)	-	0.8	-	-	-	MS
1452	α,*p*-Dimethylstyrene	0.2	-	-	0.3	-	MS
1463	1-Heptanol	-	-	-	-	0.5	MS
1493	α-Ylangene	-	-	-	-	0.7	MS
1497	α-Copaene	-	-	0.2	0.2	1.6	RRI, MS
1532	Camphor	-	-	-	0.3	-	RRI, MS
1553	Linalool	0.7	4.7	0.5	0.8	-	RRI, MS
1562	1-Octanol	-	-	-	-	6.2	MS
1586	Pinocarvone	0.3	-	0.3	1.8	-	RRI, MS
1591	Bornyl acetate	-	0.2	2.8	4.1	0.4	RRI, MS
1594	*trans*-β-Bergamotene	0.3	1.2	0.7	0.4	1.7	MS
1600	β-Elemene	13.6	7.7	12.9	5.7	-	MS
1611	Terpinen-4-ol	0.8	0.6	1.1	0.8	-	RRI, MS
1612	β-Caryophyllene	3.4	1.1	7.9	2.9	8.8	RRI, MS
1648	Myrtenal	1.1	0.9	0.7	4.0	-	MS
1661	*trans*-Pinocarvyl acetate	3.8	3.3	1.5	2.3	-	MS
1669	Sesquisabinene	-	-	-	-	1.7	MS
1670	*trans*-Pinocarveol	0.9	0.8	0.5	2.4	-	RRI, MS
1687	α-Humulene	0.8	0.4	1.4	0.6	1.0	RRI, MS
1687	Methyl chavicol	-	-	-	-	2.6	RRI, MS
1688	Selina-4,11-diene	0.4	0.3	1.1	-	-	MS
1695	(*E*)-β-Farnesene	-	-	-	-	0.4	MS
1704	Myrtenyl acetate	-	-	-	-	-	MS
1704	γ-Muurolene	-	-	0.6	0.5	2.7	MS
1706	α-Terpineol	2.4	2.5	5.1	3.9	-	RRI, MS
1719	Borneol	0.2	-	0.7	1.2	-	RRI, MS
1725	Verbenone	-	-	-	0.7	-	RRI, MS
1726	Germacrene D	-	0.3	-	-	-	RRI, MS
1740	α-Muurolene	-	-	-	-	1.6	MS
1742	Geranial	-	0.5	-	-	-	RRI, MS
1742	β-Selinene	1.5	1.2	2.9	1.6	0.9	MS
1744	α-Selinene	1.4	0.9	2.3	1.5	0.7	MS
1766	1-Decanol	-	-	-	-	3.3	MS
1773	δ-Cadinene	-	0.3	1.3	0.3	4.0	MS
1776	γ-Cadinene	-	0.1	-	0.5	2.0	MS
1784	(*E*)-α-Bisabolene	0.4	0.6	1.2	0.4	0.8	MS
1799	Cadina-1,4-diene	-	-	-	-	0.3	MS
1804	Myrtenol	1.5	1.4	0.5	2.2	-	MS
1808	Nerol	-	0.1	-	-	-	RRI, MS
1849	Calamenene	-	-	0.5	0.4	1.8	MS
1857	Geraniol	-	2.5	-	-	-	RRI, MS
1864	*p*-Cymen-8-ol	1.0	0.4	-	0.6	-	RRI, MS
1872	*cis*-Myrtanol	-	tr	-	-	-	MS
1879	*trans*-Myrtanol	-	0.2	-	-	-	MS
1941	α-Calacorene	-	-	0.2	0.2	0.9	MS
1948	*trans*-Jasmone	-	1.0	-	-	-	MS
2008	Caryophyllene oxide	3.9	0.9	1.8	7.2	1.9	RRI, MS
2029	Perilla alcohol	-	-	-	0.3	-	MS
2050	(*E*)-Nerolidol	1.3	1.7	0.6	0.4	1.2	RRI, MS
2071	Humulene epoxide-II	0.6	0.2	0.3	1.2	-	MS
2080	Junenol (=*Eudesm-4(15)-en-6-ol*)	-	0.2	-	-	-	MS
2100	Heneicosane	-	0.5	-	-	-	RRI, MS
2186	Eugenol	-	-	-	-	1.3	RRI, MS
2187	T-Cadinol	0.3	0.5	0.7	0.2	0.3	MS
2209	T-Muurolol	0.3	0.8	0.9	0.7	0.1	MS
2226	Methyl hexadecanoate	0.4	-	0.6	-	0.3	RRI, MS
2219	δ-Cadinol	-	-	0.2	-	-	MS
2255	α-Cadinol	0.3	1.3	1.4	0.7	0.3	MS
2256	Cadalene	-	-	-	-	0.7	MS
2262	Ethyl hexadecanoate	-	-	-	-	0.7	MS
2269	Guaia-6,10(14)-dien-4β-ol	0.3	-	0.6	0.7	-	MS
2273	Selin-11-en-4α-ol	1.0	1.8	4.0	1.5	-	MS
2300	Tricosane	-	0.6	-	-	-	RRI, MS
2316	Caryophylla-2(12),6(13)-dien-5β-ol (=Caryophylladienol I)	-	-	-	1.4	-	MS
2353	Chavicol	-	-	-	-	0.7	MS
2369	(2*E*,6*E*)-Farnesol	-	2.3	-	-	-	MS
2389	Caryophylla-2(12),6-dien-5β-ol (=Caryophyllenol I)	-	-	-	1.8	-	MS
2456	(Z)-9-Methyl octadecanoate (=Methyl oleate)	-	-	1.7	-	0.5	RRI, MS
2509	(Z.Z)-9,12-methyl octadecadienoate (=Methyl linoleate)	-	-	-	-	0.7	RRI, MS
2931	Hexadecanoic acid	-	-	0.5	-	2.9	RRI, MS
	Monoterpene hydrocarbons	30.9	43.8	21.6	15.2	17.7	
	Oxygenated monoterpenes	16.8	23.3	18.2	36.9	0.4	
	Sesquiterpene hydrocarbons	21.4	13.5	32.0	15.5	31.5	
	Oxygenated Sesquiterpenes	8.4	10.3	11.7	16.2	4.6	
	Fatty acids and their esters	0.4	-	2.8	-	5.1	
	Aliphatic esters	-	-	-	-	10.9	
	others	0.2	2.1	-	0.3	15.0	
	Total	78.1	93.0	86.3	84.1	85.2	

RRI: relative retention indices calculated against n-alkanes; %: calculated from FID data; tr: trace (< 0.1 %); IM: identification method based on the relative retention indices (RRI) of authentic compounds on the HP Innowax column; MS, identified based on computer matching of the mass spectra with those of the Wiley and MassFinder libraries and comparison with literature data. % calculated from FID data.; -: not detected.

**Table 2:** Toxicity of essential oils from *M.* *grandiflora* and its select pure compounds against 1-day-old *Ae. aegypti* at 24-h post-treatment.

Essential oil	LC_50_ (95%CI) *	LC_90_ (95%CI)	χ^2^	DF
Immature fruit	49.4 (39.4–64.2)	135.9 (96.5–244.2)	43.0	48
Mature fruit	48.9 (42.3–56.9)	116.9 (94.6–158.3)	83.3	48
1-Decanol	4.8 (4.2–5.5)	10.2 (8.5–13.2)	83.2	48
1-Octanol	34.3 (30.3–38.7)	63.9 (54.4–80.5)	73.7	48
Leaf	20% **			
Flower	0%			
Seed	50%			

* LC_50_ and LC_90_ values are in ppm and 95% C.I are confidence intervals. ** Leaf, flower and seeds essential oils gave only 20%, 0 and 50% mortality, respectively, at the highest dose of 125 ppm.

## Supplementary Materials

The following are available online at https://www.mdpi.com/1420-3049/25/6/1359/s1. The total ion current (TIC) chromatogram of essential oils of the leaves, flowers, immature fruits, mature fruits and seeds of *Magnolia grandiflora* are available as supporting information (Figure S1).
