# Supplementary material for: Insecticidal and Biting Deterrent Activities of Magnolia grandiflora Essential Oils and Selected Pure Compounds against Aedes aegypti
Source: Molecules. 2020 Mar 17;25(6):1359. doi: 10.3390/molecules25061359 (PMC7144101; doi:10.3390/molecules25061359)
Supplement: Supplementary file 1 [file molecules-25-01359-s001.pdf]

molecules

Supplementary Material

Insecticidal and biting deterrent activities of *Magnolia grandiflora* essential oils and selected pure compounds against *Aedes aegypti*

Abbas Ali[1,*], Nurhayat Tabanca[1,2], Betul Demirci[3], Vijayasankar Raman[1], Jane M. Budel[4], K. Husnu Can Baser[5] and Ikhlas A. Khan[1]

[1]National Center for Natural Products Research, The University of Mississippi, University, MS 38677, USA; aali@olemiss.edu (A.A.); vraman@olemiss.edu (V.R.); ikhan@olemiss.edu (I.A.K.)
[2]United States Department of Agriculture, Agricultural Research Service (USDA-ARS), Subtropical Horticulture Research Station (SHRS), Miami, Florida 33158, USA; Nurhayat.Tabanca@usda.gov (N.T.)
[3]Department of Pharmacognosy, Faculty of Pharmacy, Anadolu University, 26470 Eskisehir, Turkey; betuldemirci@gmail.com (B.D.)
[4]Departamento de Ciências Farmacêuticas, Universidade Estadual de Ponta Grossa (UEPG), Ponta Grossa, PR 84030-900, Brazil; anemanfron@hotmail.com (J.M.B.)
[5]Department of Pharmacognosy, Faculty of Pharmacy, Near East University, 99138, Nicosia, Northern Cyprus; khcbaser@gmail.com (K.H.C.B.)
*Corresponding Author:
Abbas Ali: National Center for Natural Products Research, The University of Mississippi, University, MS, 38677 USA
Email: Abbas Ali: aali@olemiss.edu; dr_aliabbas@hotmail.com

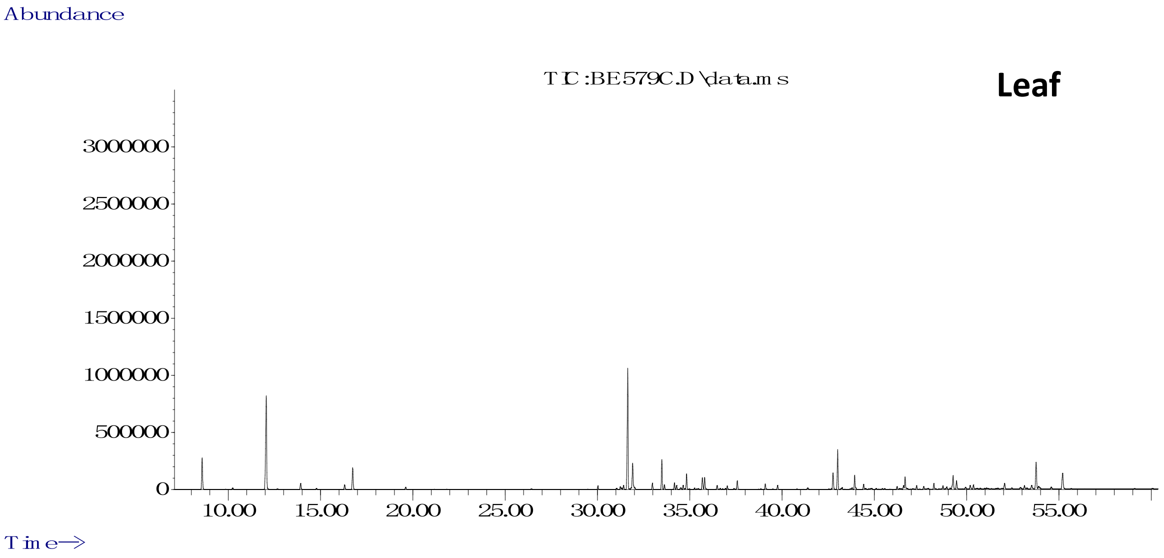

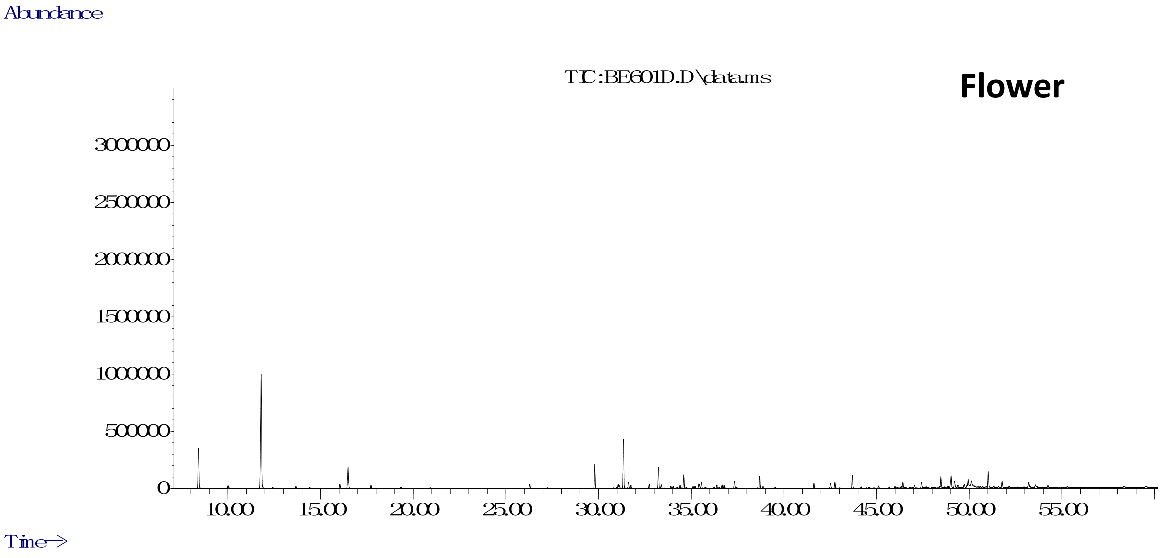
Abundance
TIC: BE601D.D\data.ms
Flower
3000000
2500000
2000000
1500000
1000000
500000
0
10.00
15.00
20.00
25.00
30.00
35.00
40.00
45.00
50.00
55.00
Time-->

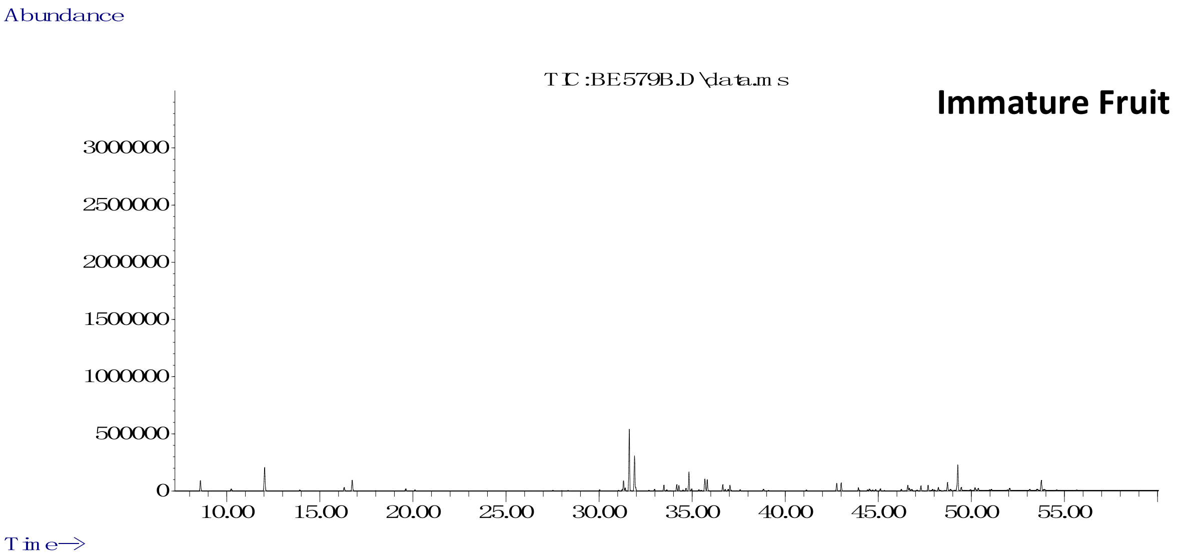
Abundance
TIC: BE579B.D\data.ms
Immature Fruit
3000000
2500000
2000000
1500000
1000000
500000
0
10.00
15.00
20.00
25.00
30.00
35.00
40.00
45.00
50.00
55.00
Time-->

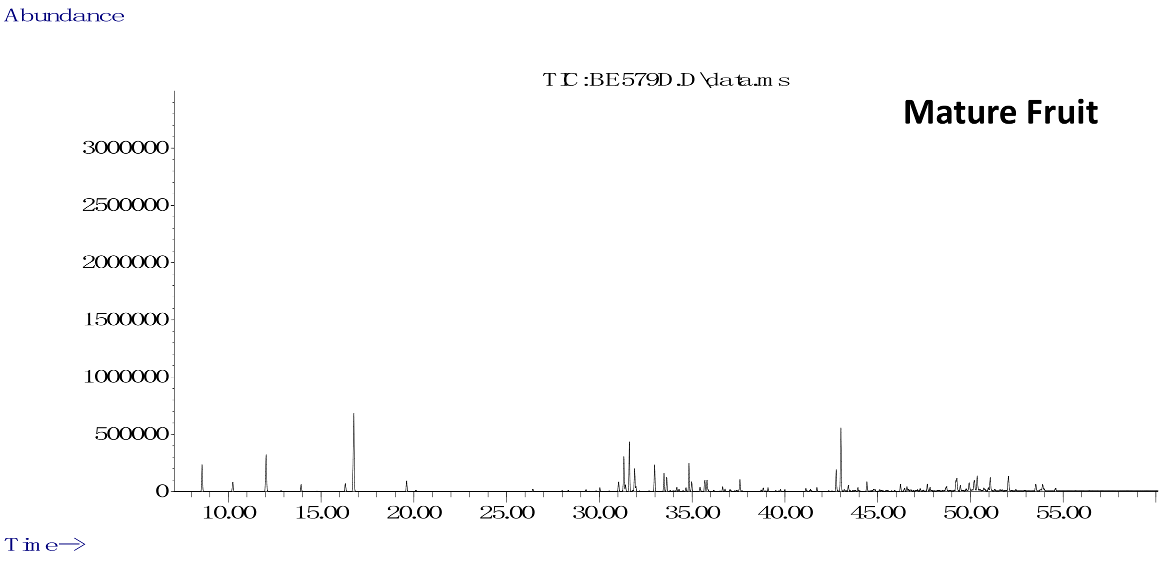
Abundance
TIC: BE579D.D\data.ms
Mature Fruit
3000000
2500000
2000000
1500000
1000000
500000
0
10.00
15.00
20.00
25.00
30.00
35.00
40.00
45.00
50.00
55.00
Time-->

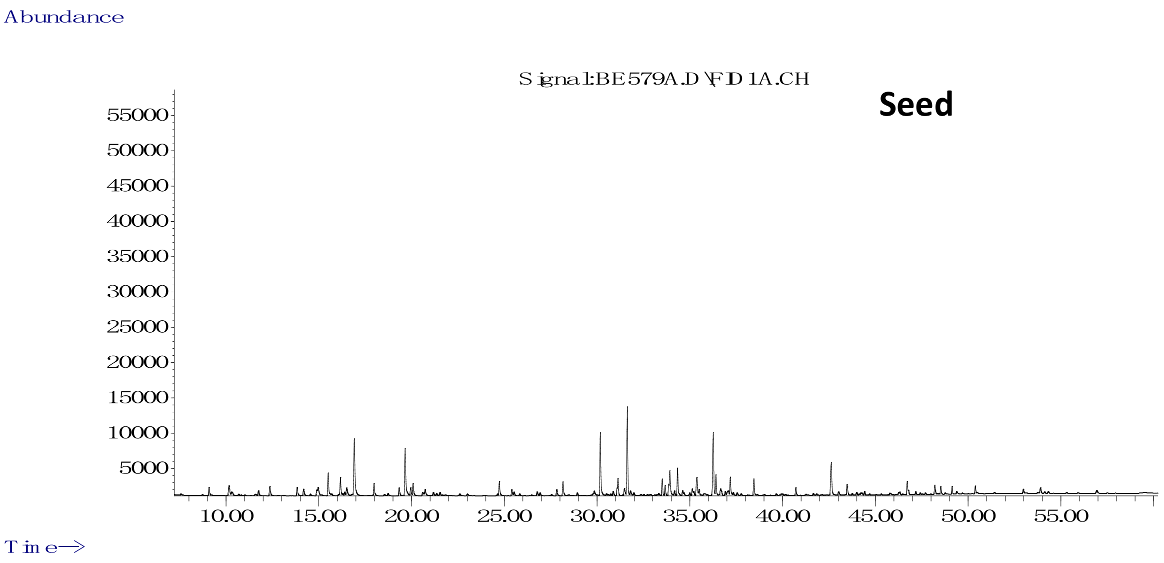

Figure S1. The total ion current (TIC) chromatogram of essential oils of the leaves, flowers, immature fruits, mature fruits and seeds of *Magnolia grandiflora*
